# QT Interval Derived Measurements in Patients with Cardiac Syndrome X Compared to Coronary Artery Disease

**DOI:** 10.3389/fphys.2016.00422

**Published:** 2016-09-21

**Authors:** Mohamed F. Lutfi

**Affiliations:** Department of Physiology, Faculty of Medicine and Health Sciences, Al-Neelain UniversityKhartoum, Sudan

**Keywords:** cardiac syndrome X, coronary artery disease, QT dispersion, JT dispersion, ventricular repolarization

## Abstract

Previous studies assessing effect of ischemia on ventricular repolarization are mostly directed toward patients with coronary artery disease (CAD); however, similar reports on cardiac syndrome X (CSX) are scarce. Whether microvascular dysfunction of CSX and ischemia induced by CAD produce comparable effect on ventricular repolarization is unclear and deserve further studies. In the present study, ECG measures of ventricular repolarization were compared between CAD and CSX patients (40 subjects in each group). Following evaluation of sociodemographic characteristics, medical and past medical history, a resting ECG was used to assess measurements of ventricular repolarization in each patient, namely, QT interval (QT), corrected QT interval (QTc), QT dispersion (QTd), corrected QT dispersion (QTcd), adjacent QT dispersion (AdQTd), QT dispersion ratio (QTdR), JT dispersion (JTd), and Corrected JT dispersion (JTcd). Results showed comparable QT intervals and QTd in CAD and CSX patients even after adjustment for the possible variations in gender, age and body mass index of the studied groups. Although JTd was increased in CSX subjects (26.6 ± 7.2 ms) compared with CAD patients (22.7 ± 6.5 ms, *p* = 0.019), statistical significance disappeared after correcting JT for variations in heart rate. QT and QTc were significantly below 440 ms in CAD as well as CSX patients (*p* < 0.001). In contrast, maximum QTd, maximum QTcd and AdQTd of CAD and CSX patients were significantly above 440 ms (*p* < 0.001). The means of JTd and JTcd were significantly above 22 ms and 24 ms respectively (*p* < 0.001, *p* = 0.001) in CSX but not CAD patients (*p* = 0.529, *p* = 0.281). The present findings clearly demonstrate comparable measures of ventricular repolarization in CAD and CSX patients and consequently an equal risk of cardiac events in both groups.

## Introduction

Presence of typical angina and positive cardiac stress tests are not necessarily due to coronary artery disease (CAD) (Levitt et al., 2013; Makharova et al., 2013). Cardiac syndrome X (CSX) is a term commonly used in the literature to describe patients with typical angina, one or more abnormal cardiac stress test(s) and normal epicardial arteries on coronary angiography (Vermeltfoort et al., 2010). Microvascular dysfunction (Panting et al., 2002) and augmented sensitivity to pain (Cannon, 1995) are the most common etiological mechanisms described in the literature for CSX. Myocardial ischemia is known to compromise ventricular repolarization putting affected patients at higher risk of ventricular arrhythmia (VA) (Ostovan et al., 2008; Çağlar et al., 2014). However, whether microvascular dysfunction of CSX and ischemia induced by CAD produce comparable effect on ventricular repolarization is unclear and deserves further studies (Tomkiewicz-Pajak et al., 2001; Alici et al., 2011). Currently, studies evaluating ventricular repolarization are typically based on parameters derived from QT or JT intervals in electrocardiography (ECG) (Tomkiewicz-Pajak et al., 2001; Yilmaz et al., 2006; Ostovan et al., 2008; Alici et al., 2011; Çağlar et al., 2014). Previous researches exploring effects of myocardial ischemia on measures of ventricular repolarization are mostly directed toward CAD patients (Higham et al., 1995; Bogun et al., 1996; Cin et al., 1997; Yilmaz et al., 2006), with only few reports on CSX (Tomkiewicz-Pajak et al., 2001; Alici et al., 2011). The present study aims to compare effects of CSX and CAD on measures of ventricular repolarization based on parameters derived from QT and JT intervals in ECG. In contrast to previous studies in the field, the present study used more comprehensive QT/JT derived measurements.

## Materials and methods

Forty patients with CAD and a similar number with CSX were recruited to the study from Cardiac Catheterization Center in El-Shaab hospital, Khartoum, Sudan. Diagnoses of CAD and CSX were based on results of coronary angiograms. Patients with stenosis/stenoses in half (or more) of the caliber of one (or more) major coronary artery/arteries were diagnosed as CAD (Mohareb et al., 2015). Alternatively, subjects with typical chest pain, positive cardiac stress test and normal coronary angiography were considered as CSX (Lee et al., 2015). All patients underwent routine echocardiography before coronary angiography. Patients with cardiomyopathy, congenital or valvular heart diseases were excluded from both groups of the study.

Following assessment of sociodemographic characteristics and past medical history, all studied subjects underwent clinical examination and ECG recording. Clinical examination included measurement of blood pressure, weight and height. Body mass index (BMI) was estimated using the equation: BMI (kg/m ^∧^2) = weight (kg)/(height (m ^∧^ 2).

A Bluetooth ECG transmitter and receiver (DM systems (Beijing) Co. limited—China) was used for ECG recording. This is a computerized resting ECG machine capable of interval/amplitudes/axis measurements and automatic interpretation. Proper function of both Bluetooth ECG transmitter and receiver for each ECG session was ensured by detecting heart rate on the screen, clean ECG signals and absence of movement artifacts. The standard 12-lead ECG recording was performed at rest, in supine position and maintained for 90 s with the following settings: 25 mm/s ECG recording speed and 1 cm/mV voltage calibration. Bluetooth ECG transmitter and receiver (BETR) software was allowed to calculate all components of QT interval and dispersion as follows:

1. **QT interval (QT)**
  QT was measured from the start of the QRS complex to the end of T wave (if termination of T wave was clearly identified) or the nadir between the U and T-waves (if U wave was obvious). Using BETR software, the maximum (Max QT), minimum (Min QT), and average QT intervals of all leads were measured in milliseconds (ms).
2. **Corrected QT interval (QTc)**
  QTc is QT interval adjusted for the possible influences of heart rate. QTc was measured using Bazett's formula ($QTc\;=\;QT/\sqrt{RR}$). Same like QT intervals, the maximum (Max QTc), minimum (Min QTc), and average QTc intervals of all leads were measured in ms.
3. **QT dispersion (QTd)**
  QTd was estimated by the formula: *QTd* = *Max QT* − *Min QT* in any of the ECG leads. Using BETR software, maximum (Max QTd), and average QTd were measured from all leads.
4. **Corrected QT dispersion (QTcd)**
  QTcd was estimated by the formula: *QTcd* = *Max QTc* − *Min QTc* in any of the ECG leads. Same like QTd, maximum (Max QTcd), and average QTc dispersion were measured from all leads.
5. **Adjacent QT dispersion (AdQTd)**
  AdQTd was measured by estimating the maximum difference between Max QT and Min QT in two adjacent standard precordial ECG leads.
6. **QT dispersion ratio (QTdR)**
  QTdR was estimated by the formula: *QTdR* = *QTd*×100/*RR*, where RR is the period between two successive R waves. QTdR was expressed as percentage.
7. **JT dispersion (JTd)**
  JTd was estimated by the formula: *JTd* = *Max JT* − *Min JT*, where Max JT and Min JT were the maximum and minimum JT interval respectively. JT interval was measured from the J-point (i.e., the end of QRS) and the end of T wave (if T wave had clearly identified termination) or the nadir between the U and T-waves (if U wave was obvious).
8. **Corrected JT dispersion (JTcd)**
  JTcd was estimated by the formula: *JTcd* = *Max JTc* − *Min JTc*, where Max JTc and Min JTc were the maximum and minimum JTc interval respectively. JTc interval was calculated using the equation *JTc* = *QTc* − *QRS*.

### Data analysis

Statistical Package for the Social Sciences (SPSS) for windows (Version 16; Chicago, IL, USA) was used for statistical analysis. Normal distribution of the studied variables were ensured using Shapiro–Wilk test. Comparable distribution of gender among studied group was assessed by Chi^2^ test. Possible significant differences in the means/standard deviations (SD) of age, BMI, QT, QTc, QTd, QTcd, AdQTd, QTdR, JTd, and JTcd between CAD and CSX patients were evaluated by unpaired Student's *T*-test. Using a general linear model, gender, age and BMI for each subject were introduced as covariates while comparing all measurements of ventricular repolarization between the studied groups. QT, QTc, JT, JTc, and all dispersion measurements of CAD and CSX were also compared with cutoff reference values using one sample T-test. The cutoff reference values used were 440 ms for QT and QTc (Christensen et al., 2000), 50 ms for QTd, QTcd, and AdQTd (Macfarlane et al., 1998; Christensen et al., 2000), 6% for QTdR (Nussinovitch et al., 2012), 22 ms for JTd (Duraković et al., 2001; Nussinovitch et al., 2012) and 24 ms for JTcd (Duraković et al., 2001; Nussinovitch et al., 2012). A value of *P* < 0.05 was considered significant.

### Ethics

The ethical clearance of this study was approved by the ethics review committee at Faculty of Medicine, Khartoum University, Sudan. All studied subjects signed a written consent before being enrolled in the study.

## Results

The basic characteristics of the studied groups are given in Table 1.

**Table 1:** **Characteristics of the studied CSX and CAD patients**.

	**CSX *N* = 40**	**CAD *N* = 40**	***P***
Age (years) (*M ± SD*)	51.0 ± 16.9	58.7 ± 10.3	0.017^*^
Male gender (*N*(%))	17 (42.5%)	25 (62.5%)	0.073
BMI (kg/m$\hat{2}$) (*M ± SD*)	29.7 ± 5.2	26.2 ± 4.3	0.002^*^
SBP (mmHg) (*M ± SD*)	133.8 ± 20.1	127.7 ± 22.5	0.214
DBP (mmHg) (*M ± SD*)	79.4 ± 12.3	78.6 ± 12.9	0.789
Diabetes mellitus (*N*(%))	8 (20%)	18 (45%)	0.017^*^
Hypertension (*N*(%))	17 (42.5%)	20 (50%)	0.501
Smoking (*N*(%))	17 (42.5%)	20 (50%)	0.434

BMI, body mass index; CAD, coronary artery disease; CSX, cardiac syndrome X; DBP, diastolic blood pressure; M, mean; N, number; SBP, systolic blood pressure; SD, standard deviation; %, percentage;

* Statistically significant.

As shown in Table 2, all components of QT intervals and QTd were comparable in CAD and CSX patients even after adjustment for the possible variations in gender, age and BMI of the studied groups. Although JTd was higher in CSX subjects (26.6 ± 7.2 ms) compared with CAD patients (22.7 ± 6.5 ms, *p* = 0.019), statistical significance disappeared after correcting JT for variations in heart rate (JTcd = 28.5 ± 7.9 ms in CSX subjects vs. 25.5 ± 8.5 ms in CAD patients, *p* = 0.163).

**Table 2:** **Comparisons of QT derived values between CSX and CAD patients**.

	**CSX *N* = 40 *M ± SD***	**CAD *N* = 40 *M ± SD***	***p***	
			**Non-adjusted**	**Adjusted for age and BMI**
Max QT (ms)	399.9 ± 39.6	393.7 ± 34.9	0.468	0.260
Min QT (ms)	329.7 ± 37.8	330.0 ± 33.4	0.976	0.651
Average QT (ms)	382.6 ± 38.4	378.8 ± 33.7	0.643	0.288
Max QTc (Bazett) (ms)	427.1 ± 35.9	438.7 ± 48.7	0.239	0.207
Min QTc (Bazett) (ms)	352.3 ± 37.4	367.5 ± 48.3	0.131	0.147
Average QTc (Bazett) (ms)	408.6 ± 34.1	421.4 ± 48.2	0.183	0.221
Max QTd (ms)	68.5 ± 18.0	62.9 ± 19.1	0.185	0.176
Average QTd (ms)	49.4 ± 16.6	43.5 ± 15.9	0.117	0.286
Max QTcd (ms)	73.1 ± 18.5	69.5 ± 20.4	0.420	0.521
Average QTcd (ms)	52.7 ± 17.2	48.1 ± 17.2	0.240	0.640
AdQTd (ms)	68.5 ± 18.0	62.9 ± 19.1	0.185	0.176
QTd Ratio (%)	5.7 ± 1.9	5.4 ± 2.0	0.503	0.863
JTd (ms)	26.6 ± 7.2	22.7 ± 6.5	0.016^*^	0.019^*^
JTcd (ms)	28.5 ± 7.9	25.5 ± 8.5	0.120	0.163

AdQTd, adjacent QT dispersion; CAD, coronary artery disease; CSX, cardiac syndrome X; JTcd, Corrected JT dispersion; JTd, JT dispersion; M, mean; Max, maximum; Min, minimum; ms, millisecond; QT, QT interval; QTc, corrected QT interval; QTcd, corrected QT dispersion; QTd, QT dispersion; QTdR, QT dispersion ratio; SD, standard deviation,

* Statistically significant.

Maximum, average and minimum values of QT and QTc were significantly below 440 ms in CAD as well as CSX patients (Table 3). In contrast, maximum QTd, maximum QTcd, and AdQTd of CAD and CSX patients were significantly above 50 ms (Table 3). The means of JTd and JTcd were significantly above reference values in CSX but not CAD patients (Table 3).

**Table 3:** **Comparisons of QT derived values of CSX and CAD patients with the cutoff reference values**.

	**CSX *N* = 40 *M ± SD***	**CAD *N* = 40 *M ± SD***	**Cutoff reference value**	***p***	
				**CSX vs. RV**	**CAD vs. RV**
Max QT (ms)	399.9 ± 39.6	393.7 ± 34.9	440 ms	< 0.001^*^	< 0.001^*^
Min QT (ms)	329.7 ± 37.8	330.0 ± 33.4	440 ms	< 0.001^*^	< 0.001^*^
Average QT (ms)	382.6 ± 38.4	378.8 ± 33.7	440 ms	< 0.001^*^	< 0.001^*^
Max QTc (Bazett) (ms)	427.1 ± 35.9	438.7 ± 48.7	440 ms	0.028^*^	0.876
Min QTc (Bazett) (ms)	352.3 ± 37.4	367.5 ± 48.3	440 ms	< 0.001^*^	< 0.001^*^
Average QTc (Bazett) (ms)	408.6 ± 34.1	421.4 ± 48.2	440 ms	< 0.001^*^	0.025^*^
Maximum QTd (ms)	68.5 ± 18.0	62.9 ± 19.1	50 ms	< 0.001^Δ^	< 0.001^Δ^
Average QTd (ms)	49.4 ± 16.6	43.5 ± 15.9	50 ms	0.822	0.018^*^
Maximum QTc d (ms)	73.1 ± 18.5	69.5 ± 20.4	50 ms	< 0.001^Δ^	< 0.001^Δ^
Average QTcd (ms)	52.7 ± 17.2	48.1 ± 17.2	50 ms	0.323	0.501
AdQTd (ms)	68.5 ± 18.0	62.9 ± 19.1	50 ms	< 0.001^Δ^	< 0.001^Δ^
QTdR (%)	5.7 ± 1.9	5.4 ± 2.0	6%	0.280	0.066
JTd (ms)	26.6 ± 7.2	22.7 ± 6.5	22 ms	< 0.001^Δ^	0.529
JTcd (ms)	28.5 ± 7.9	25.5 ± 8.5	24 ms	0.001^Δ^	0.281

AdQTd, adjacent QT dispersion; CAD, coronary artery disease; CSX, cardiac syndrome X; JTcd, Corrected JT dispersion; JTd, JT dispersion; M, mean; Max, maximum; Min, minimum; ms, millisecond; QT, QT interval; QTc, corrected QT interval; QTcd, corrected QT dispersion; QTd, QT dispersion; QTdR, QT dispersion ratio; SD, standard deviation;

* Significantly less than the cutoff reference value;

Δ significantly more than the cutoff reference value.

## Discussion

The present results revealed four main findings: firstly, QT and QTc values were comparable in CAD and CSX patients and confined to the normal physiological range in either group. Secondly, QTd, QTcd, and AdQTd values were not statistically different between CAD and CSX patients; however, these measurements exceeded the upper limit of normal ranges in either group. Thirdly, JTd was higher in CSX subjects compared with CAD patients but failed to achieve similar result when correcting JT for variations in heart rate (JTcd). Lastly, JTd and JTcd were both significantly above reference values in CSX but not CAD patients.

The present findings are comparable with earlier reports (Higham et al., 1995; Bogun et al., 1996; Cin et al., 1997; Tomkiewicz-Pajak et al., 2001; Yilmaz et al., 2006; Alici et al., 2011; Lee et al., 2015; Mohareb et al., 2015), although QT-derived parameters evaluated in previous studies are generally less comprehensive compared to our study. Alici et al. (2011) compared components of QT and QTd of 66 patients with CSX with a control group of 39 patients with typical angina, normal coronary angiographies but negative exercise treadmill testing (ETT). Results revealed comparable Max QT, Min QT and QTd in CSX and the control group at rest. However, these measurements were significantly higher in CSX subjects than in the control group during exercise. In addition, ST segments were significantly more depressed in CSX patients with QTd = 60 ms than those with QTd < 60 ms. In another study, QTc and QTcd were measured in 20 women with CAD, 19 women with CSX and 14 healthy control women at rest and during exercise (Tomkiewicz-Pajak et al., 2001). There were no significant differences in QTd between different studied groups at rest. In contrast, QTcd was significantly greater in CAD and CSX than in healthy control group, both at rest and during exercise. The study suggested that QTd and QTcd are not effective tools to differentiate between women with CAD from those with CAD. In a comparable study, Lee and his colleagues assessed effects of postural changes on QTd in patients with CAD and CSX (Lee et al., 2015). According to Lee et al, there were significant differences in QTd or QTcd between CAD and CSX on upright standing, but not at baseline or exercise. Lee et al concluded that CSX patients had a higher QTcd in response to standing from the supine position probably due to enhanced sympathetic activity. Based on the results of the three studies stated above, it is evident the values of QTd and QTcd tend to be comparable, but equally higher in either CAD or CSX patients compared with the healthy controls. Our results give further support for this hypothesis because comparable QTd and QTcd were demonstrated in CAD or CSX patients, yet both measurements were above the upper limit of normal.

Studies assessing other components of ventricular repolarization like AdQTd, QTdR, JTd, and JTcd are scarce and mostly targeted CAD rather than CSX patients. To our knowledge, the present study is the first to explore these parameters in CSX patients. Our results demonstrate comparable AdQTd, QTdR, and JTcd in CSX and CAD patients; however, JTd was greater in the first group. The present data also demonstrated higher JTd and JTcd in CSX compared to expected reference values; nonetheless, the studied CAD patients failed to show the same trend. These findings do agree with many previous studies (Furniss and Campbell, 1995; Macfarlane et al., 1998; Christensen et al., 2000; Duraković et al., 2001; Nussinovitch et al., 2012); but not others (Zita et al., 2004). The conclusion drawn by Bogun et al. (1996) that CAD patients with high QT dispersion are at higher risk of VA was partly based on evaluation of AdQTd in the studied groups. In a comparable study, AdQTd was used by Ostovan et al. (2008) to evaluate the susceptibility of patients with acute myocardial infarction (AMI) to VA. Ostovan data suggested that AdQTd ≥ 45 ms on the second day following AMI is a good indicator of the likelihood to develop VA. In cases with chronic cardiac ischemia, QTdR seems to increase proportional to severity of coronary atherosclerosis in CAD patients (Yilmaz et al., 2006). This fact is supported by a recent study that confirmed the tendency of QTdR values to upsurge significantly from single vessel toward three vessels CAD (Çağlar et al., 2014). According to some reports, QTdR is superior to QTd and QTcd in assessing risk of ventricular fibrillation (Higham et al., 1995) and probably other types of VA (Cin et al., 1997) in CAD patients. A study designed to evaluate ventricular repolarization in CAD showed no difference in JTd when women without coronary artery stenoses were compared with one or two vessel CAD at rest (Zita et al., 2004). However, JTd > 33 ms at the peak of exercise achieved the best diagnostic value compared to JT and ST products. In another study, measurements of ventricular repolarization were assessed in 17 patients who died because of arrhythmia induced by CAD and 51 matched survivors (Zareba et al., 1994). Results of multivariate analyses strongly suggested prolonged JTd and JTcd as independent risk factors of arrhythmic cardiac death.

The comparable QT-derived values in CSX and CAD patients suggest comparable effects of these diseases on the ventricular recovery times (Malik and Batchvarov, 2000). Coronary microvascular dysfunction is common in CSX patients and may be part of a more generalized vascular disorder involving other organs in the body (Cannon, 1995; Masci et al., 2005). This hypothesis is augmented by abnormal levels in nitric oxide and endothelin-1 (Piatti et al., 2003) and higher prevalence of migraine (Nakamura et al., 2000) in CSX patients. Coronary macro- and micro-vascular dysfunction induces myocardial ischemia in CAD and CSX respectively, which in turn prolong ventricular recovery time (VRT). The disturbed VRT explains why most measurements of dispersions are comparably increased in CAD and CSX compared with normal reference values. Actually, all measurements of dispersions evaluated in the present study were higher in CSX compared to CAD patients; however, only JTd achieved statistical significance. This interesting finding should motivate researchers to investigate if CSX subjects are at higher risk to develop VA compared to CAD patients.

Worth mentioning, although QT derived parameters are frequently referred to as the repolarization parameters; these measures actually reflect both activation and recovery of ventricular myocardium. In the ECG, the QT interval coincides with depolarization and repolarization of ventricular myocardium and consequently its derived parameters are influenced by problems affecting both ventricular phases (Li et al., 2001; Kawasaki et al., 2003). However, since ventricular repolarization occupies most of the duration of the ventricular action potential and almost all refractory period, this phase seems to possess higher influences on the ventricular recovery time and pathogenesis of arrhythmia (Zabel et al., 1998; Xia et al., 2005).

Limitations of this study include absence of a healthy control group. Precise exclusion of CAD and CSX in the control group may necessitate performance of coronary angiography and cardiac stress tests. Ethically, both procedures are difficult to perform for patients without complaints, especially elder individuals. Consequently, measurements of ventricular repolarization were interpreted by making a comparison with cutoff reference values. Noteworthy, values of various types of QT and JT dispersions showed wide overlap between healthy subjects and diseased patients (Davey et al., 1994; Shah et al., 1998; Duraković et al., 2001; Nussinovitch et al., 2012). The previously reported variations in the normal physiological range of different types of QT and JT dispersions are possibly due to influences of gender (Macfarlane et al., 1998), age (Macfarlane et al., 1998; Tran et al., 2001), and BMI (Mangoni et al., 2003) in these measurements. In the present study, the results were interpreted according to the AHA/ACCF/HRS recommendations for the standardization and interpretation of the electrocardiogram: part IV (Rautaharju et al., 2009) and other comparable reports (Macfarlane et al., 1998; Christensen et al., 2000; Duraković et al., 2001; Nussinovitch et al., 2012). Although adjustment for gender, age and BMI were also considered when comparing measurements of ventricular repolarization between CSX and CAD patients, the present study did not adjust for possible influences of medical therapy offered to patients and external atmospheric effects, which could also have effects on repolarization.

## Conclusions

The present data clearly demonstrate comparable measures of ventricular repolarization in CAD and CSX patients. However, all QT-derived measurements of dispersion exceeded the upper limit of normal ranges in either group. Interestingly, both JTd and JTcd were significantly above reference values in CSX but not CAD patients. These findings suggest that the ventricular recovery time and consequently the risk of VA in CSX is comparable or even more than CAD. Further studies that also consider other electrophysiological parameters like duration of QRS and T waves, transmural repolarization gradients, ventricular late potential and follow-up for potential cardiac events are desirable to support the present findings.

## Author contributions

All work was conducted by MFL (study design, data collection, analysis, interpretation, and draft writing).

### Conflict of interest statement

The author declares that the research was conducted in the absence of any commercial or financial relationships that could be construed as a potential conflict of interest.
